# Microstructure and chemical data of the thermoelectric ZnSb material after joining to metallic electrodes and heat treatment

**DOI:** 10.1016/j.dib.2017.09.023

**Published:** 2017-09-15

**Authors:** Safdar Abbas Malik, Le Thanh Hung, Ngo Van Nong

**Affiliations:** Department of Energy Conversion and Storage, Technical University of Denmark, Risø Campus, 4000 Roskilde, Denmark

## Abstract

The data presented in this article are related to the research article entitled: “Solder free joining as a highly effective method for making contact between thermoelectric materials and metallic electrodes” (Malik et al., 2017) [1]. This article presents microstructure obtained by scanning electron microscopy (SEM) and chemical analysis by energy dispersive X-ray spectroscopy (EDX) point measurements of the thermoelectric ZnSb legs after joining to metallic electrodes using solder (Zn-2Al) and free-soldering methods.

**Specifications Table**

**Table:** 

Subject area	*Material Science*
More specific subject area	*Thermoelectric Energy Conversion*
Type of data	*Table, Image (microscopy)*
How data was acquired	*SEM/EDX analysis*
Data format	*Raw, Analyzed*
Experimental factors	*The ZnSb legs prepared by Spark Plasma Sintering technique from reaction of elemental commercial powders. The surfaces of the leg were polished and cleaned before joining.*
Experimental features	*The quality of material after joining with metallic electrodes was examined.*
Data source location	*Technical University of Denmark, Risø Campus, 4000 Roskilde, Denmark.*
Data accessibility	*The data presented in this article are accessible within this article.*

**Value of the data**

• This data elaborates the importance of solder free joining method for making good contacts in thermoelectric devices.
• The data presented in this article shows detailed microstructure and EDX analysis of ZnSb material after joining and heat treatment.
• This data allows other researchers to compare the conventional joining method with new solder-free joining method.

## 1 Data

**Fig. 1:**
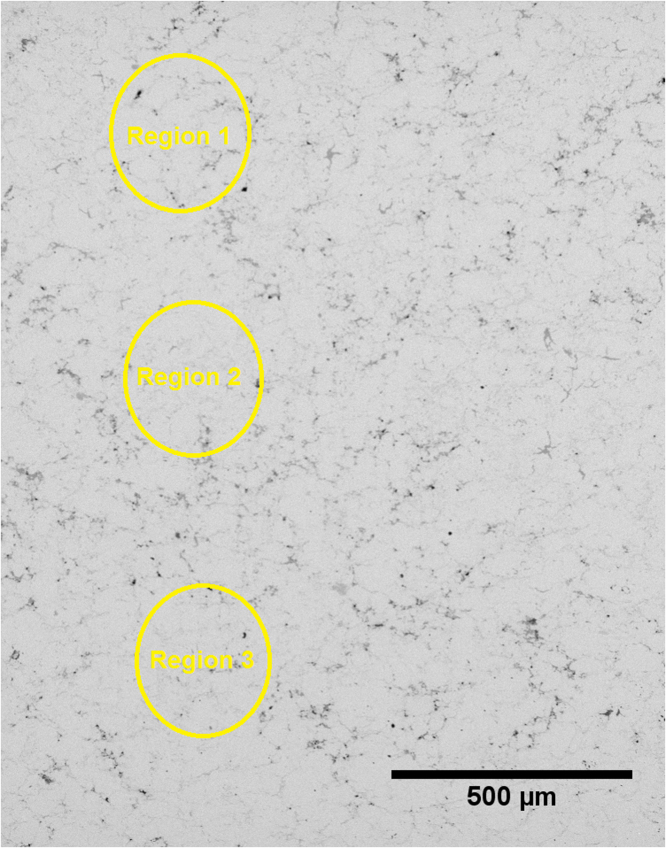
SEM micrograph and selected EDX point measurements of the ZnSb leg after joining to metallic electrodes using Zn − 2Al solder.

**Fig. 2:**
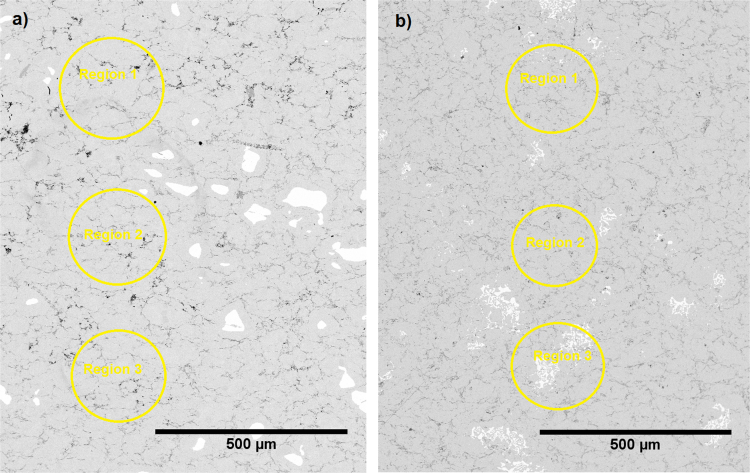
SEM micrograph and selected EDX point measurements along the ZnSb leg after solder-free joining to Ni electrode with (a) Ti and (b) Cr as interconnecting agents.

**Fig. 3:**
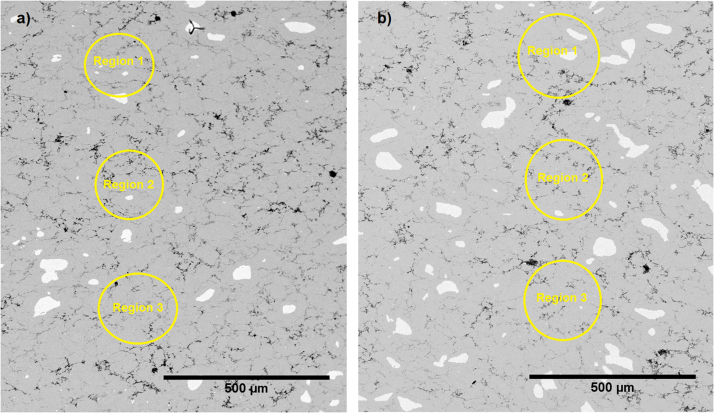
SEM micrograph of the ZnSb leg after solder-free joining and heat treatment for 30 hours at 400 °C with (a) Ti and (b) Cr as interconnecting agents.

The following data provides information on the SEM images and EDX analysis along the thermoelectric ZnSb legs. The Fig. 1, Fig. 2, Fig. 3 show micrographs of the ZnSb legs after joining and heat treatment. Table 1, Table 2, Table 3 present the concentration ratio of Zn:Sb at selected regions along the leg.

**Table 1:** Typical EDX point measurements along the ZnSb leg shown in Fig. 1.

**% Ratio**	**Region 1**	**Region 2**	**Region 3**	**Average**
Zn:Sb	56.5:43.0	55.3:44.7	56.2:43.4	56:44

**Table 2:** Typical EDX point measurement along the ZnSb legs shown in Fig. 2.

**% Ratio**	**Region 1**	**Region 2**	**Region 3**	**Average**
(a) Zn:Sb	48.9:51.1	47.9:52.1	48.9:51.1	48.5:51.5
(b) Zn:Sb	49.8:50.2	50.5:49.5	52.3:47.7	50.8:49.2

**Table 3:** Typical EDX point measurement along the ZnSb legs shown in Fig. 3.

**% Ratio**	**Region 1**	**Region 2**	**Region 3**	**Average**
(a) Zn:Sb	50.9:49.4	50.8:49.2	51.4:48.6	∼51:49
(b) Zn:Sb	50.1:49.9	51.9:48.1	51.5:48.5	∼51:49

### 1.1 After conventional joining with solder

Fig. 1 presents a typical SEM micrograph of the ZnSb leg after conventional joining using Zn − 2Al solder alloy. The chemical analysis of selected EDX point measurements along the leg is presented in Table 1. The average ratio of Zn:Sb is 56:44.

### 1.2 After solder-free joining

Fig. 2 presents SEM micrographs of the ZnSb legs after solder-free joining with (a) Ti and (b) Cr as interconnecting agents. The EDX point measurements on selected regions are presented in Table 2. The average Zn:Sb ratios are 48.5:51.5 for (a) and 50.8:49.2 for (b).

Fig. 3 shows SEM micrograph of the ZnSb leg after solder-free joining and heat treatment for 30 hours at 400 °C with (a) Ti and (b) Cr as interconnecting agents. The typical EDX point measurements are given in Table 3.

## 2 Experimental design, materials and methods

ZnSb ingots used for this study were provided by TEGnology AS, Denmark. ZnSb legs with dimension of 3×3×3 mm^3^ were cut to join with metallic electrodes (Ni, Ag) using two methods: the conventional with solder and a solder-free method [1]. The joining were performed in the temperature range of 400–450 °C under a pressure of 3 MPa for 30 min. Heat treatment of the joint parts was carried out at 450 °C for 30 h. The SEM images and EDX point measurements along the ZnSb leg after joining were carried out in a Hitachi TM3000 scanning electron microscope.
