# Supplementary material for: Microstructure and chemical data of the thermoelectric ZnSb material after joining to metallic electrodes and heat treatment
Source: Data Brief. 2017 Sep 15;15:97–101. doi: 10.1016/j.dib.2017.09.023 (PMC5614718; doi:10.1016/j.dib.2017.09.023)
Supplement: Supplementary file 1 — Transparency document [file mmc1.docx]

**CONFLICT OF INTEREST AND AUTHORSHIP CONFIRMATION**

**PLEASE CHECK THE FOLLOWING AS APPROPRIATE.**

All authors have participated in (a) conception and design, or analysis and interpretation of the data; (b) drafting the article or revising it critically for important intellectual content; and (c) approval of the final version.

The Article I have submitted to the journal for review is original, has been written by the stated authors and has not been published elsewhere.

The Images that I have submitted to the journal for review are original, was taken by the stated authors, and has not been published elsewhere.

This manuscript has not been submitted to, nor is under review at, another journal or other publishing venue.

The authors have no affiliation with any organization with a direct or indirect financial interest in the subject matter discussed in the manuscript

The below authors have affiliations with organizations with direct or indirect financial interest in the subject matter discussed in the manuscript:

**Safdar Abbas Malik, Le Thanh Hung, Ngo Van Nong**
